# The emerging importance of skull-brain interactions in traumatic brain injury

**DOI:** 10.3389/fimmu.2024.1353513

**Published:** 2024-04-11

**Authors:** Grant W. Goodman, Patrick Devlin, Bryce E. West, Rodney M. Ritzel

**Affiliations:** Department of Neurology, McGovern Medical School, The University of Texas Health Science Center at Houston, Houston, TX, United States

**Keywords:** TBI - traumatic brain injury, bone marrow, skull & brain, neuroinflammation, Myelopoiesis

## Abstract

The recent identification of skull bone marrow as a reactive hematopoietic niche that can contribute to and direct leukocyte trafficking into the meninges and brain has transformed our view of this bone structure from a solid, protective casing to a living, dynamic tissue poised to modulate brain homeostasis and neuroinflammation. This emerging concept may be highly relevant to injuries that directly impact the skull such as in traumatic brain injury (TBI). From mild concussion to severe contusion with skull fracturing, the bone marrow response of this local myeloid cell reservoir has the potential to impact not just the acute inflammatory response in the brain, but also the remodeling of the calvarium itself, influencing its response to future head impacts. If we borrow understanding from recent discoveries in other CNS immunological niches and extend them to this nascent, but growing, subfield of neuroimmunology, it is not unreasonable to consider the hematopoietic compartment in the skull may similarly play an important role in health, aging, and neurodegenerative disease following TBI. This literature review briefly summarizes the traditional role of the skull in TBI and offers some additional insights into skull-brain interactions and their potential role in affecting secondary neuroinflammation and injury outcomes.

## Introduction

Traumatic Brain Injury (TBI) plays a significant role in morbidity and mortality within the United States, encompassing a range of injuries affecting brain function due to an external force to the skull (1). Approximately 2.8 million head injuries occur each year in the United States alone, with skull fracture occurring in 28-37% of those diagnosed with TBI (2, 3). The burden of TBI is significant, with fatalities associated with TBI estimated to number more than 69,000 annually and 3.2-5.3 million individuals suffering from lasting disability in the US (4, 5). TBI predominates among men, young children, and elderly populations, with incidence rates influenced by socioeconomic factors (6). Following either penetrating or blunt, non-penetrating traumas, TBI leads to symptoms such as altered consciousness, amnesia, confusion, headache, or dizziness (7). Severe complications include intracranial hemorrhage (ICH), heightened intracranial pressure (ICP), brain edema, and seizures (8). The initial trauma is often termed the primary injury, leading to the secondary injury or the pathophysiological responses to trauma (9). The secondary injury includes neuroinflammatory processes, excitotoxicity, and eventual cell death (10). Current treatment strategies for TBI primarily focus on the secondary injury mechanisms following brain injury. Most of these involve relieving elevated ICP via surgical intervention, anti-inflammatory medication, cranial temperature modulation, or elevation of the head (11).

Penetrating TBI involves foreign object intrusion into brain tissue, often due to injuries caused by projectiles like gunshot wounds (7). In contrast, blunt, non-penetrating TBI results from direct head impact or rapid head acceleration and deceleration without direct contact (7). Blunt non-penetrating TBI frequently leads to a contrecoup brain injury, causing brain contusion on the opposite side to the external force (12). This phenomenon arises due to the sudden movement of denser cerebrospinal fluid (CSF) towards the skull impact point, displacing the brain towards the opposite side of the skull and causing the contrecoup injury (13). The health and structural integrity of the skull as a living tissue, is thus directly and critically involved in determining the severity of TBI. However, until recently, the involvement of skull bone marrow cells, including hematopoietic cells, bone-modifying osteocytes, and other stromal cells, has been long overshadowed by the evolving neuropathology seen in the brain.

The International Classification of Diseases, Tenth Revision, Clinical Modification (ICD-10-CM) diagnostic codes for TBI include skull fracturing (7). However, TBI severity is typically classified as mild, moderate, or severe based on the Glasgow Coma Scale (GCS), which evaluates eye-opening, motor, and verbal responses (14). The GCS does not account for skull fracturing in TBI. Notably, skull fractures are evident in approximately 5% of mild TBI cases and up to 50% of severe TBI cases, correlating with worse TBI prognoses (2, 15). Non-penetrating TBI accounts for 90% of skull fractures, with the remaining 10% originating from penetrating TBI (16). Disparities between these two evaluations, and more, may result in improper treatment plans and poorer outcomes.

While research has extensively explored the secondary brain injury following TBI, the biological role of the skull in mitigating or exacerbating the pathophysiological response remains unclear. Initially regarded as a static protective structure for the brain and meninges, recent studies have uncovered a significant role for the skull bone marrow (BM) as a distinctive site housing immune cells crucial for sensing and participating in central nervous system (CNS) inflammation (17). Moreover, direct connections between the skull and brain allow skull BM-derived immune cells to migrate into brain tissue after injury (18). This review aims to synthesize current literature on experimental TBI models, the inflammatory response post-TBI, and the skull’s involvement in TBI. Furthermore, we aim to evolve the view of the skull as not just an inert protective casing for the brain, but as a dynamic tissue that is actively involved in the brain’s response to trauma.

## The skull and TBI

The human neurocranium is a composite bone structure composed of two compact cranial tables enclosing a cancellous intermediary layer of the skull, termed the diploe (19). Importantly for the discussion of brain-skull immune dynamics, the diploe serves as the primary reservoir of skull-derived bone marrow. Studies investigating the relative force capacity of each of the skull’s layers have found that it is this composite sandwich structure that accounts for the human skull’s impressive durability, rather than the strength of any individual layer (19). As skull thickness may be a significant protective modifier in TBI and skull fracture risk, apart from impact velocity, one of the most important predictors of blunt force TBI severity is impact location. Bone thickness within the cranium ranges significantly, even within the same individual bone plate (19). Unlike other bone structures, cranium bone thickness does not appear to decrease in an age-dependent manner, but it does exhibit the same trend of reduction in flexibility (20). Currently, the interaction between age and sex on cranial thickness and strength remains unclear (20). It is worth noting here that while skull fracture can occur in severe TBI and contribute to post-injury fragility, moderate TBI has been shown to cause an increase in cranial thickness which may be protective against cranial fracture in cases of repeated TBI (21).

Furthermore, while the skull serves an invaluable protective role against the mechanical forces that result in brain injury, it also contributes to TBI through the contact forces imposed on the brain during coup and contrecoup injury, as well as through the sheering forces induced between superficial brain areas and the bony protrusions of the frontal and temporal internal cranial fossae (22). Although the direct impact of the brain within the cranial vault mediates primary injury, the skull also provides a means for therapeutic intervention via craniotomy to release ICP. Additionally, the vascular channels acting as an interface between the skull and meninges have been proposed as a potentially novel route of drug delivery across the BBB, with intraosseous administration of drug compounds reaching 10- to 100-fold greater penetration into the brain parenchyma compared to systemic administration (23). Emerging data also indicate that skull bone health may be critical for normal brain function, especially in the context of repetitive mild concussion or age-specific TBI (24). Skull-targeted therapies may be in the nascent phase of preclinical development but have strong potential to attenuate the local inflammatory environment or prevent bone fracturing and dural bleeding.

## Acute inflammatory response to TBI

During the secondary phase of injury, there are numerous mechanisms to promote the inflammatory response following TBI, including apoptotic cascades, reactive oxygen species (ROS) generation, increased BBB permeability, and mitochondrial dysfunction (25–27). Damage-Associated Molecular Patterns (DAMPs) like ATP, Heat-Shock Proteins (HSPs), and High Mobility Group Box 1 (HMGB1) are released from neurons and glial cells, activating innate immune receptors on macrophages, dendritic cells, and glial cells (28). This activation prompts microglia, the resident macrophages of the CNS, to clear debris and intensify inflammation by releasing pro-inflammatory cytokines (29, 30). These cytokines, including IL-1B and IL-6, recruit neutrophils to the affected area (31, 32). Elevated serum IL-6 levels in severe TBI patients have been found to correlate with poorer outcomes, suggesting intensified inflammation results in more prominent pathology (33). Within 24 hours of TBI, microglia release nitric oxide (NO), ROS, IL-1B, and IL-6, enhancing the recruitment and differentiation of monocytes into tissue macrophages (34, 35). The nucleotide-binding oligomerization domain-like receptor pyrin domain-containing-3 (NLRP3) inflammasome activation by innate immune regulator, nuclear factor- κB (NFκB), in microglia and astrocytes contributes to a pro-inflammatory environment via caspase cleavage and IL-1B and IL-18 secretion (36). In TBI, microglial-secreted IL-6 and NFκB upregulate the aquaporin (AQP4) water channel in endothelia, which is known to promote brain edema (32, 37, 38). Chronic microglial activation following TBI has been posited as one of the drivers of long-term cognitive dysfunction seen in TBI, with one study of former National Football League players showing increased binding of the microglia-associated inflammatory marker, translocator protein (TSPO), in brain regions including the supramarginal gyrus and amygdala (39). In addition to the increase in inflammatory signaling in the brain parenchyma, the meninges is rapidly gaining interest as an area of immune activation in the context of TBI (40, 41). Indeed, in recent neuroimaging studies, patients with mild TBI show meningeal abnormalities that may indicate areas of increased inflammation (42).

Lymphoid immune cells have also been demonstrated to have significant involvement in the inflammatory response to TBI. Following TBI, CD4+ T cells differentiate into T-regulatory (Treg) and T-helper (Th) subsets Th1, Th2, and Th17 (43). Th1 and Th17 play strong pro-inflammatory roles in the ensuing immune response. Th1 cells release IL-2, interferon-gamma (IFN-y), and tumor necrosis factor-alpha (TNF-a) to activate macrophages and increase BBB permeability (44, 45). Human CD4+ Th17 cells, via IL-17 and IL-22 secretion, disrupt the BBB and cause CNS inflammation (46). CD4+ Th2 cells secrete neuroprotective cytokines IL-4 and IL-5, promoting anti-inflammatory processes (47). Th cell polarization is driven by microglial secretion of the chemokine CXCL10, promoting Th1 cell infiltration and overall brain inflammation (48). A previously understudied, but critically important player in this neuroinflammatory response to TBI is the immune cell-rich lymphoid tissue found in the skull.

## Immune contribution of the skull

**Figure 1:**
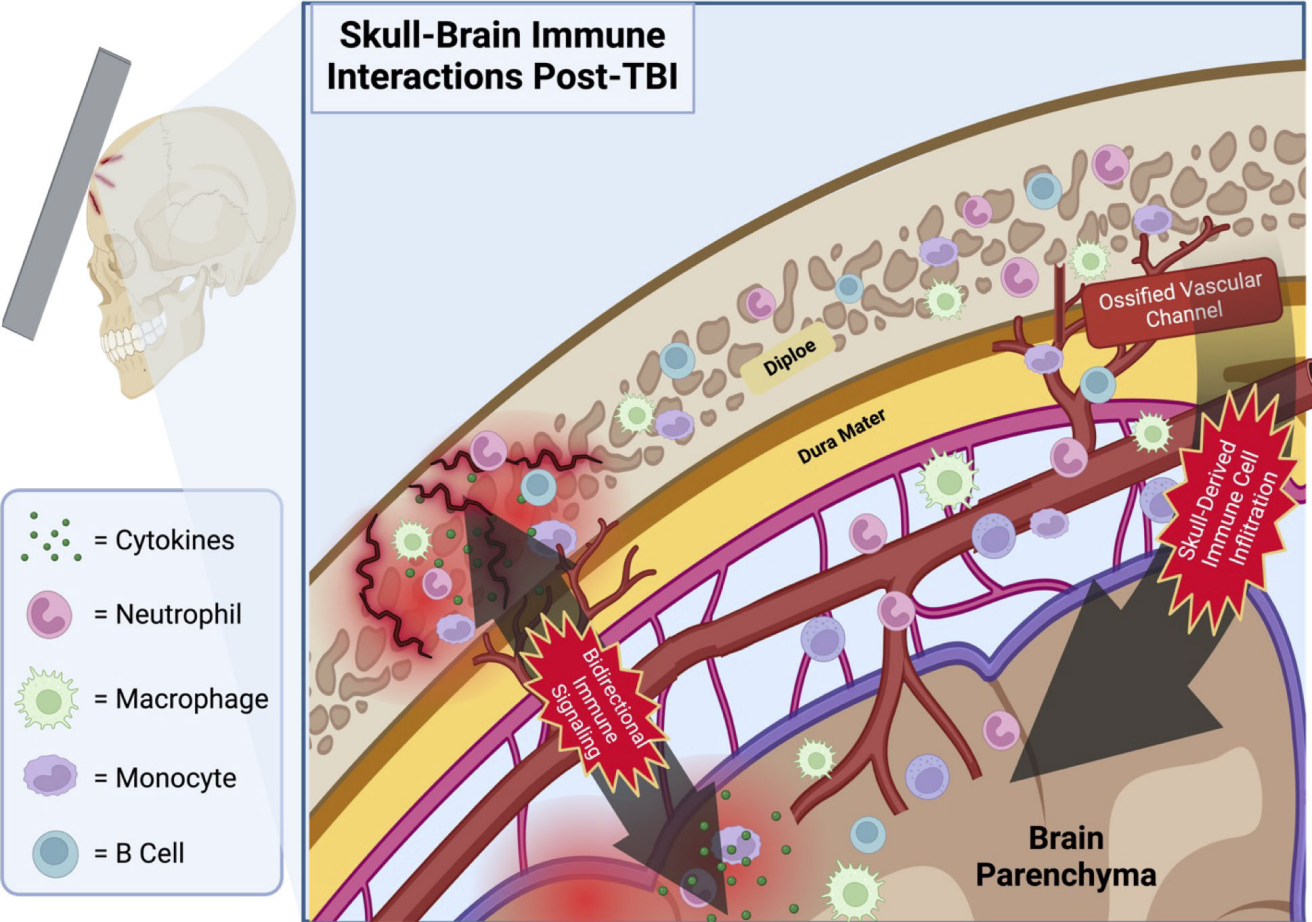
Proposed schematic depicting the infiltration of skull-derived immune cells into the brain parenchyma via ossified vascular channels following TBI with microfractures. Immune cells located in the calvarial bone marrow of the skull’s diploe have a direct portal to the meninges via the shared vasculature penetrating this cancellous bone tissue. Note the bidirectional travel of immune signaling molecules through these same channels. Illustration created with BioRender.com.

Although the skull has been largely overlooked in neurotrauma research, it contains millions of immune cells in a proximal location to the brain and is thus poised to act as a significant modulator of CNS function via regulation of skull-meningeal trafficking (**Figure 1**). The immune compartment in the diploe of the skull calvarium may also play a pivotal role in the immune activation following TBI via its direct access to arachnoid CSF absorption sites in the meninges through short ossified paravascular channels (49, 50). Differentiated from other bone marrow compartments by its distinct transcriptomic and proteomic profile, calvarium bone marrow has been identified as a major source of immune cell recruitment to the CNS following injury to the brain (51). Multiple studies have demonstrated infiltration of skull-derived immune cells into the CNS following brain injury, indicating a previously uninvestigated role of skull bone marrow in contributing to the inflammatory response to TBI (52). The bone marrow is a complex environment containing various cell types of endothelial cells, osteocytes, and matrix components that support the immune system and hematopoietic stem cells (HSCs) (53–55). Within this environment, HSC proliferation and differentiation play a pivotal role in immune responses, giving rise to both lymphoid and myeloid cell lineages (56, 57). In fact, during the acute inflammatory response to TBI, there is a well-characterized shift in HSC differentiation towards the myeloid cell lineage in the nearby skull marrow (58). Furthermore, damage to the skull has been shown to directly influence immune dynamics in the brain, with murine models of a weight-drop TBI demonstrating increased inflammation-related gene expression of TNF-α and TIMP-1 in the brain tissue of mice with skull fractures compared to those without (59). Mice with skull fractures also had significantly worse Neurological Severity Scores (NSS) than those without, and skull fracture severity correlated positively with NSS (59). It is not clear whether TBI-associated with or even without skull fractures resulted in greater leukocyte infiltration into the brain. Notably, murine models of CNS trauma demonstrate the presence of myeloid progenitors in the meninges during neuroinflammation, which are otherwise absent without trauma (60, 61). Combined with findings that indicate myeloid cell migration from both the skull and peripheral blood, evidence strongly suggests a role for skull bone marrow in the initial immune response to TBI (17, 62).

One of the main vehicles proposed to enable the transmigration of immune cells into the brain parenchyma CSF has also been implicated in signaling myeloid cell movement from the skull BM to the meninges by accessing skull diploe and mobilizing immune cells following insult (63). Additionally, these vascular portals between the CNS and the skull enable traffic in both directions, with immune factors from the CNS traveling to bone marrow niches in the skull to drive HSC differentiation and recruitment (64). The greatest source of this heterogeneity arises from differing genetic profiles of the resident myeloid and B cells. Multiple studies have demonstrated the presence of calvaria-derived neutrophils, monocytes, macrophages, and B cells in the meninges and brain parenchyma (17). While the precise role of the skull-derived hematopoietic cells in TBI remains uncertain, the existing literature indicates a clear mechanism for immune cell infiltration from the skull’s BM into the brain parenchyma following injury.

Importantly, the skull-meninges interface described here is bidirectional. This is supported by studies showing that CSF can exit into skull bone marrow to instruct hematopoietic response to neuroinflammatory conditions, including infection, stroke, and multiple sclerosis (64, 65).Additionally, this skull-meninges connection may not only be a route of traffic for immune cells, but also secretory factors which may further modulate neuroinflammation following TBI. Unfortunately, there is to date little research directly investigating the changes to calvaria bone marrow composition following TBI. While there is no longer doubt as to the neuroinflammatory contribution of skull-derived immune cells located in the dura following brain injury, whether the neuroimmune response is preferential to migration from local bone marrow sites over circulating, bloodborne immune cells is a question which requires further investigation (17).

## Glymphatic-meningeal lymphatic circulation

An important consideration to account for in the discussion of CSF circulatory dynamics between the skull and the meninges is TBI’s impact on paravascular glymphatic and perivascular lymphatic function in the meninges and brain. Normally responsible for the clearance of neurotoxic substances such as DAMPs, Aβ aggregates, tau proteins, and alpha-synuclein aggregates, the glymphatic and lymphatic systems in the brain play a vital role in recovery from TBI and are a burgeoning area of research in the field (66). TBI has been shown to significantly impact the rate of flow in these drainage systems. Following injury, the rate of paravascular drainage is slowed by as much as 60%, causing a subsequent reduction in the movement of interstitial fluid through the brain and an increase in protein accumulation in the parenchyma (67). Furthermore, it has recently been shown that enhancement of glymphatic-lymphatic drainage via a “nano-plumber” technology results in a significant improvement in neurological function in rodents following TBI (68). This treatment works by returning injured tissue to a homeostatic microenvironment via dampening the local microglial response and recovering healthy vascular and glymphatic-meningeal lymphatic circulation to support the clearance of noxious material and pro-inflammatory immune cells. The disruption to paravascular drainage in the parenchyma may well extend to the meningeal-skull marrow paravascular interface following TBI, however, the consequence of such a disruption for the function of immune signaling and migration between the skull and meninges is currently unknown.

## Skull fracturing and morphological adaptation to TBI

Despite the dissipative effect on impact energy that may occur when the skull fractures in response to mechanical trauma, one of the greatest clinical risk factors for mortality and worsened outcomes in severe TBI is the presence of fractures to the skull vault or skull base (69). In the weight-drop animal model of TBI, skull fractures are reported to induce a significant increase in the inflammatory response compared to TBI mice without fractures (59). Due to the heterogeneity of cranial bone thickness and geometry, the likelihood of fracture varies significantly based on which region of the skull an impact occurs. Microfracturing is rarely examined in imaging studies but may be a key predictor of more serious fracturing with repeated head impacts. Recent studies investigating impact force dynamics on the human skull suggest that the temporal region is the area most susceptible to fracture, largely due to its thinner diploe compared to more resilient areas of the skull in the frontal, parietal, and occipital regions (19). The skull also appears to exhibit mechanosensitive adaptations to physical impact, increasing in thickness in the area exposed to the impact force in a dose-dependent manner. Preliminary research shows that this bone anabolic effect may be mediated in part by the cannabinoid-1 receptor (70). Furthermore, the underlying meninges appears to respond in a similarly localized fashion, mounting a dynamic transcriptomic response that is exacerbated with age (21). As with the potential role of the skull’s immune response in modulating TBI outcome, the influence of changes to skull morphology at both the macroscopic and cellular levels following TBI may serve as another path to explore potential therapeutic interventions for recovery from TBI.

## Experimental models for TBI

Due to the heterogeneity of injuries associated with TBI, there are a number of different experimental animal models to mimic different aspects of both primary and secondary TBI progression and pathology. The current prevailing models in the TBI literature include controlled cortical impact (CCI), weight drop-impact acceleration injury, fluid percussion injury, blast injury, and penetrating ballistic-like brain injury (71). Although each of these models comes with its own advantages and disadvantages, experimental paradigms such as the weight drop-impact acceleration model and other closed-head models of TBI are better suited to study the skull-brain axis of immunity due to their comparatively less invasive manipulation of the calvarium. However, evidence of leukocyte infiltration is absent in most weight drop models of mild concussion. Injury models, such as CCI, that involve direct manipulation of the skull tissue prior to injury induction, most often in the form of a partial craniotomy over the impact location, may serve to obscure the potential influence of proximal skull tissue on injury progression and recovery following TBI. This is evidenced clinically by the high proportion of patients who undergo craniotomy experiencing fever in the post-operative period (72). Furthermore, as CT analysis of skull fracturing is commonly used for clinical diagnosis of TBI (73), models that incorporate this aspect of TBI pathology will be especially useful in future research investigating the influence of the skull in TBI secondary injury and recovery.

## Conclusion

As one of the leading causes of neurological disability in the world, the study of TBI pathophysiology and its potential therapeutic interventions is a matter of pressing importance. Despite this, investigation of nearby tissues with preferential access and influence over immune dynamics within the brain is an area of relative neglect in TBI literature until recently. With the discovery of vascular channels by which the immune compartments of the brain and skull may communicate, there is a greater impetus than ever before to expand research seeking to investigate the role of this connection and how it may be incorporated into future treatment approaches.

## Author contributions

GG: Writing – original draft. PD: Writing – original draft. BW: Writing – original draft. RR: Conceptualization, Funding acquisition, Supervision, Writing – review & editing.
